# Investigation on the Quality Factor Limit of the (111) Silicon Based Disk Resonator

**DOI:** 10.3390/mi9010025

**Published:** 2018-01-22

**Authors:** Xin Zhou, Dingbang Xiao, Qingsong Li, Qian Hu, Zhanqiang Hou, Kaixuan He, Zhihua Chen, Chun Zhao, Yulie Wu, Xuezhong Wu, Ashwin Seshia

**Affiliations:** 1 College of Mechatronics Engineering and Automation, National University of Defense Technology, Changsha 410073, China; xz387@cam.ac.uk (X.Z.); liqingsong@nudt.edu.cn (Q.L.); huqian@nudt.edu.cn (Q.H.); houzhanqiang@nudt.edu.cn (Z.H.); zhchen@nudt.edu.cn (Z.C.); ylwu@nudt.edu.cn (Y.W.); xzwu@nudt.edu.cn (X.W.); 2 Nanoscience Centre, University of Cambridge, Cambridge CB3 0FF, UK; cz319@cam.ac.uk (C.Z.); aas41@eng.cam.ac.uk (A.S.); 3 State Key Laboratory of ASIC and System, School of Microelectronics, Fudan University, Shanghai 200433, China; 17112020020@fudan.edu.cn; 4 East China Institute of Photo-Electronic IC, Bengbu 233042, China

**Keywords:** Microelectromechanical systems, quality factor, thermoelastic dissipation, clamping loss, disk resonator, gyroscope

## Abstract

Quality factor is one of the most important parameters for a MEMS resonator. Most MEMS resonators are dominated by thermoelastic dissipation (TED). This paper demonstrates that the TED in a disk resonator that is made of (111) single-crystal silicon is surpassed by clamping loss. The stiffness-mass decoupling design method, combined with reducing the beam width, was used to engineer high *Q*_TED_. Experiments show that *Q* of the (111) disk resonator have an upper boundary that is determined by the clamping loss caused by the unbalanced out-of-plane displacement. The origin of the out-of-plane displacement is explained by theory and simulation.

## 1. Introduction

Microelectromechanical systems (MEMS) based resonators have wide applications, such as chemical or biological sensing [1,2], wireless filters [3,4], frequency references [5], inertial sensing [6,7,8,9], and many other applications [10,11]. One of the most important parameters for MEMS resonator is the quality factor (*Q*) defined as the ratio of the totally stored energy to the dissipated energy per vibration cycle, because *Q* is directly related to the sensitivity of these MEMS devices. Many mechanisms have been found to contribute to the overall damping of the MEMS resonators. Some well-known damping mechanisms include: air damping [12,13], surface loss [14,15], clamping loss [16,17], and thermoelastic dissipation (TED) [18,19]. Different kinds of dissipation mechanisms are treated as paralleling dampers, and the overall *Q* is determined by *Q*^−1^ = ∑*Q_i_*^−1^, where *i* labels the different mechanisms.

For most of the well-designed, well-fabricated, and well-packaged MEMS resonators, TED is the dominating damping factor at room temperature [20,21,22]. In this study, the dissipation of a high performance disk-like resonator is elaborated. Owing to its high thermal stability, large modal mass, and many other advantages, the disk resonator is successfully used for high performance MEMS gyroscopes [6,7,21]. A lot of researches have been implemented to improve the performance of disk resonator [6,7,23,24,25,26,27,28,29,30]. One promising technique is to decouple the stiffness and mass of the disk resonator by adding lumped masses to the frame structure [28,29]. This method is ideal for enhancing the *Q*_TED_ of the disk resonator [30]. This method does not contradict with other *Q*-enhancing method [22,24]. The can be combined together to engineer a better resonator. In this paper, we demonstrate that the TED in a disk resonator is greatly reduced. The clamping loss surpasses TED, becoming the dominant damper. Another research also indicates that the clamping loss is the next main damping factor after the TED is mitigated [31].

## 2. Device Description

**Figure 1:**
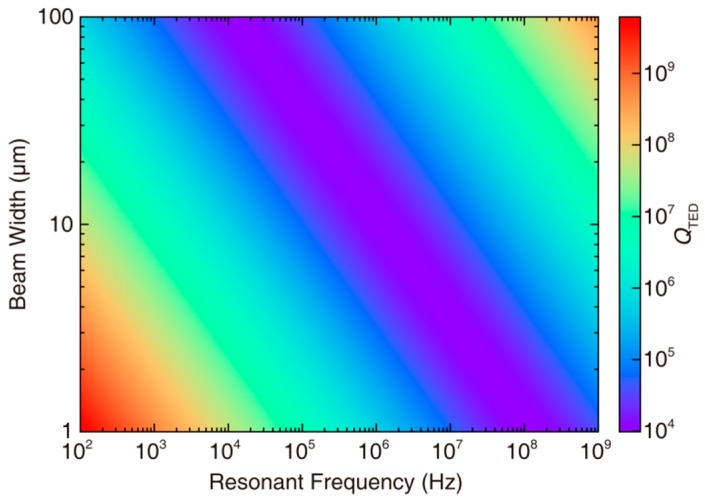
*Q*_TED_ of the single-crystal-silicon resonators based on Zener’s model as a function of resonant frequency and beam width. The left side of the “valley” is in isothermal mode, and the right side of the “valley” is in adiabatic mode.

TED is one of the most important damping factors of micromechanical resonators. Strain field in the deformed mechanical resonator will lead to a temperature gradient, which will then result in an irreversible heat flow. The energy dissipation caused by this conversion from mechanical energy to heat is defined as the TED. Zener’s standard model [25,26] is the most widely used theoretical model for TED, which is given by [18]

(1) $$Q_{\mathrm{TED}}=\frac{C_{V}}{E\alpha^{2}T_{0}}\frac{1+{\left(\omega_{0}/\omega_{\mathrm{Relax}}\right)}^{2}}{\omega_{0}/\omega_{\mathrm{Relax}}}$$

(2) $$\omega_{\mathrm{Relax}}=\frac{\chi \pi^{2}}{b^{2}}$$

where *C_V_* is the heat capacity of the solid. *E* is the Young’s modulus. *α* is the coefficient of thermal expansion. *T*_0_ is the nominal average temperature. *ω*_0_ and *ω*_Relax_ are the resonant frequency and the thermal relaxation rate, respectively. *χ* is thermal diffusivity of the solid and *b* is the width of the strained beam. *Q*_TED_ is a function of resonant frequency and beam width, as shown Figure 1. When *ω*_0_ is close to *ω*_Relax_, the damping reaches the maximum. The condition of *ω*_0_ << *ω*_Relax_ is defined as isothermal-mode, which is the left side of the “valley” in Figure 1. Conversely, condition of *ω*_0_ >> *ω*_Relax_ is defined as adiabatic-mode, which is the right side of the “valley” in Figure 1.

**Figure 2:**
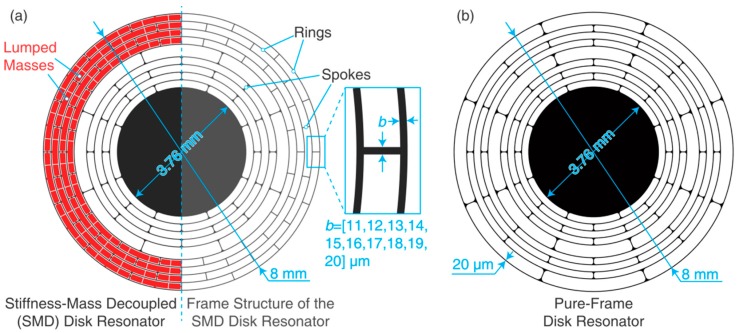
(**a**) Schematic of the stiffness-mass decoupled disk resonator. Beam width *b* is altered from 11 μm to 20 μm. (**b**) Schematic of a pure-frame disk resonator.

In this study, disk resonators based on the stiffness-mass decoupling (SMD) design method are developed. The disk resonator is made up of nine concentric rings with an outer diameter of 8000 μm. The outer four layers of rings or the rest of the inner rings are interconnected with 16-fold or eight-fold spokes, respectively. Those rings and spokes have an identical width. The outer rings and spokes are added with lumped silicon mass pieces. The silicon mass pieces with dimensions of 160 μm × 150 μm × *l* (*l* = 3 mm–4 mm) are connected to the frame structure using thin beams. The whole structure is suspended on a central anchor with a diameter of 3.760 mm. The layout of the stiffness-mass decoupled disk resonator is shown in Figure 2a. The effective stiffness *k*_eff_ is mainly provided by the frame and the effective mass *m*_eff_ is mainly provided by the lumped masses. Thus, the stiffness and the mass are decoupled. It should be noted that the amount of the lumped mass should be carefully considered, because too low a resonant frequency might be detrimental for a MEMS gyroscope.

The proposed SMD disk resonator works in the isothermal mode. Adding additional lumped masses can greatly reduce the resonant frequency, thus seperate the resonant frequency from the thermal relaxation rate. Compared to the pure-frame structure, the SMD resonator can provide much higher *Q*_TED_ [28]. Besides, the beam width has been altered to further manipulate the *Q*_TED_. SMD disk resonators with beam widths *b* ranging from 11 μm to 20 μm were engineered. For comparison, one pure-frame disk resonator is also designed, as shown in Figure 2b. The structural parameters of this resonator is similar to the SMD resonator. Its beam width is designed to be 20 μm.

**Figure 3:**
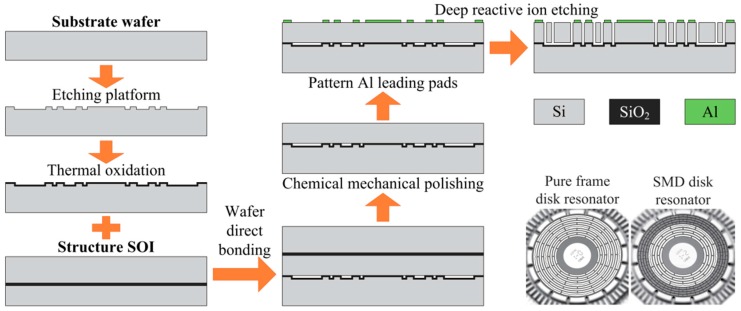
Fabrication process of the disk resonators. The photographs of a fabricated SMD disk resonator and a pure frame disk resonator are also shown.

The designed disk resonators were fabricated using a conventional micromachine process, as depicted in Figure 3. A 500 μm thick (100) single-crystal silicon wafer is used as the substrate wafer. The substrate wafer was etched to create some 5 μm high platforms for bonding. The etched substrate wafer was then thermally oxidized. A silicon-on-insulator (SOI) wafer is used to provide a 150 μm thick (111) structure layer. The structure SOI was bonded with the thermal oxidized substrate wafer using an aligned wafer direct bonding process. The auxiliary handle layer of the SOI was removed by a chemical–mechanical polishing process. Then, the structure layer is patterned with Al leading pads. Lastly, the resonator is fabricated using a deep reactive ion etching process. A fabricated SMD disk resonator and a pure frame disk resonator are also shown in Figure 3.

## 3. Characterization

**Figure 4:**
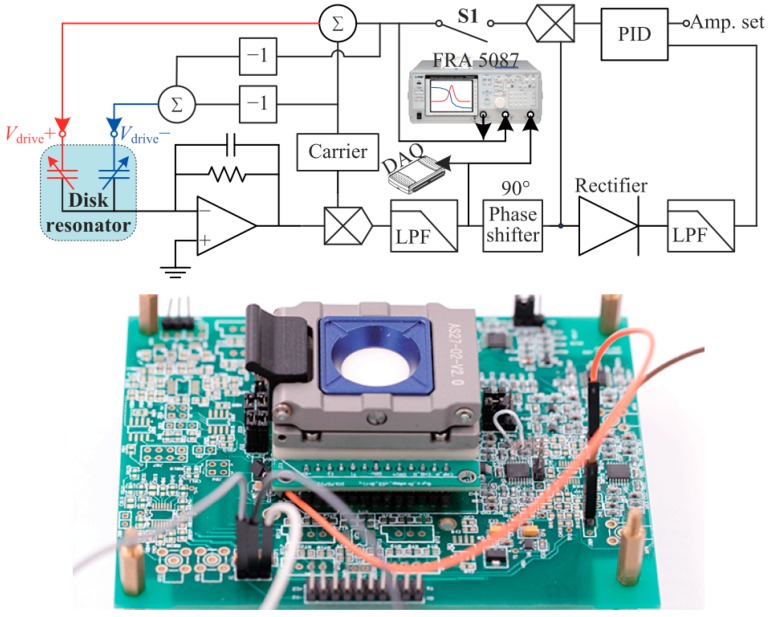
Schematic diagram of the circuit for spectrum and ring-down experiments (**up**) and the testing circuit board (**down**).

**Figure 5:**
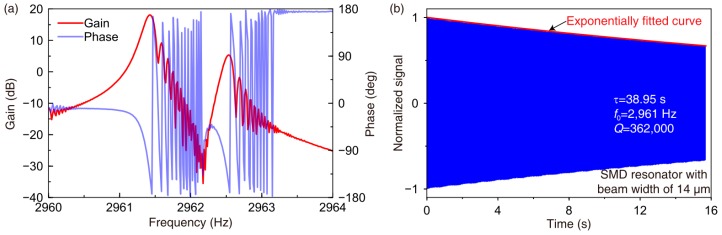
(**a**) Typical frequency response and (**b**) ring-down signal of the *n* = 2 wineglass mode of a SMD disk resonator with beam width of 14 μm.

The fabricated disk resonator is diced, attached to a ceramic leadless chip carrier, and then wire-bonded. A ring-down experiment was implemented to characterize the *Q* and decaying time constant *τ*. The spectrum method is used to find the *n* = 2 wine-glass modes. The schematic diagram of the experimental circuit is shown in Figure 4. The devices were tested in a vacuum chamber to minimize air damping. The spectrum test is used to obtain the resonant frequency. First, switch S1 was turned off. A sweeping frequency AC signal generated by a frequency response analyzer (NF FRA 5087) was applied on the Spectrum Input port in the circuit. The signal was modulated by a high-frequency carrier signal in order to reduce the low-frequency noise of the front end. The output signal was demodulated and then put in the FRA 5087 from the output port of the circuit. A typical frequency response of the SMD disk resonator is shown in Figure 5a. The frequency was swept upward. Due to the long decaying time constant and the small sweeping time interval, there were some echo signals on the right side of the peaks. Then the ring-down method was implemented to obtain the decaying time constant. First, switch S1 was turned on, and the disk resonator was originally actuated at resonance using self-oscillation. The amplitude of the driving mode was controlled using a proportion-integration-differentiation (PID) controller. Then, the actuation was stopped by turning off S1. The decaying signal in the output port of the circuit was recorded by a data acquisition (DAQ) system. The recorded decaying signal was then filtered, enveloped, and normalized. *τ* was calculated by fitting the normalized envelope with the function *A* × e^(−*t*/*τ*)^. *Q* can be calculated based on *Q* = *τω*_0_/2. A typical ring-down result of the SMD disk resonator is shown in Figure 5b.

## 4. Results and Discussion

**Figure 6:**
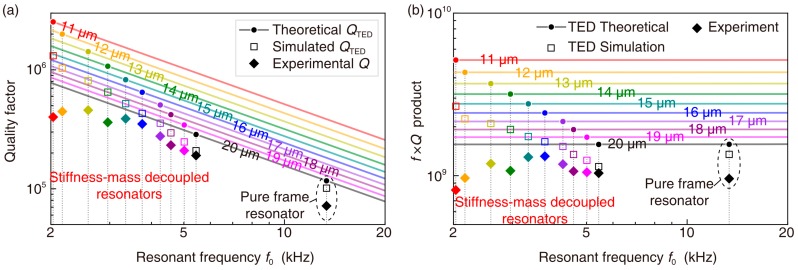
(**a**) Experimental *Q*s, simulated *Q*_TED_s, and theoretical *Q*_TED_s of the SMD disk resonators with beam width ranging from 11 μm to 20 μm and those of the pure frame disk resonator with beam width of 20 μm. (**b**) *f* × *Q* products obtained by TED theory, TED simulation, and Experiment for the SMD disk resonators with beam width ranging from 11 μm to 20 μm and those of the pure frame disk resonator with beam width of 20 μm.

The *Q*_TED_ of each kind of disk resonator was simulated using COMSOL. The TED simulation concerns the thermoelastic dynamics and thermal diffusion phenomenon. The experimental *Q*s and simulated *Q*_TED_s are illustrated in Figure 6a. The theoretical *Q*_TED_s based on Zener’s model are also included. Furthermore, the corresponding *f* × *Q* products are illustrated in Figure 6b as well. The effectiveness of the *Q*_TED_ enhancement of the stiffness-mass decoupling method is demonstrated by comparing the 20-μm pure-frame resonator with the 20-μm SMD resonator. When decreasing the beam width of the SMD resonator, *Q*_TED_ is increased, whereas *Q* stops increasing when it reaches the value around 450,000. This interesting phenomenon indicates that TED is surpassed by another damping mechanism when *Q* stops increasing. The reduction of the *f* × *Q* product when the beam width is smaller than 14 μm can also support this hypothesis.

The quality factor induced by the secondary damping mechanism *Q*_other_ is estimated to ranging from 577,000 to 1,890,000. A reasonable hypothesis is that this secondary damping is the clamping loss. The unbalanced out-of-plane displacement in the *n* = 2 wine-glass mode of the disk resonator that is made of (111) silicon is demonstrated here, which has not been cognized before. This unbalanced out-of-plane displacement may produce considerable clamping loss. The (111) silicon was believed to be a good material for symmetrical MEMS devices, because the Young’s modulus, Poisson’s ratio, and shear modulus are isotropic on (111) silicon [32]. However, there is a coupling between the in-plane and the out-of-plane deformation. The relationship between the stress tensor and the strain tensor of (111) silicon in Cartesian coordinate system is given by [33].

(3) $$\left[\begin{array}{c} \sigma_{xx}\\ \sigma_{yy}\\ \sigma_{zz}\\ \tau_{yz}\\ \tau_{zx}\\ \tau_{xy} \end{array}\right]=\left[\begin{array}{cccccc} {\bar{c}}_{11} & {\bar{c}}_{12} & {\bar{c}}_{13} & 0 & {\bar{c}}_{15} & 0\\ {\bar{c}}_{12} & {\bar{c}}_{11} & {\bar{c}}_{13} & 0 & -{\bar{c}}_{15} & 0\\ {\bar{c}}_{13} & {\bar{c}}_{13} & {\bar{c}}_{33} & 0 & 0 & 0\\ 0 & 0 & 0 & {\bar{c}}_{44} & 0 & -{\bar{c}}_{15}\\ {\bar{c}}_{15} & -{\bar{c}}_{15} & 0 & 0 & {\bar{c}}_{44} & 0\\ 0 & 0 & 0 & -{\bar{c}}_{15} & 0 & {\bar{c}}_{66} \end{array}\right]\left[\begin{array}{c} \varepsilon_{xx}\\ \varepsilon_{yy}\\ \varepsilon_{zz}\\ \gamma_{yz}\\ \gamma_{zx}\\ \gamma_{xy} \end{array}\right]$$

The subscript of the stiffness matrix are defined as: 1 is *xx*, 2 is *yy*, 3 is *zz*, 4 is *yz*, 5 is *zx*, and 6 is *xy*.

**Figure 7:**
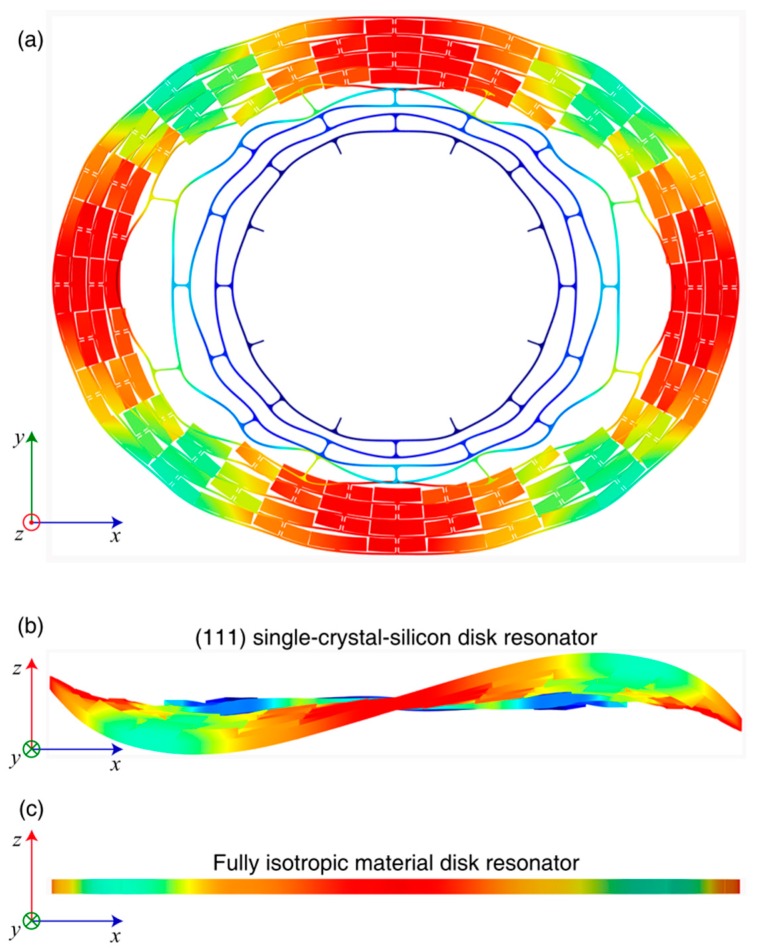
(**a**) Top view of the *n* = 2 wine-glass mode of the SMD disk resonator; (**b**) Side view of the SMD disk resonator that is made of (111) silicon. The scale of the out-of-plane deformation is amplified by 50 times; (**c**) Side view of the SMD disk resonator that is made of a fully isotropic material.

Compared with the stiffness matrix of the (100) silicon, the stiffness matrix of the (111) silicon have coupling term ${\bar{c}}_{15}$. The normal in-plane strain *ε_xx_* and *ε_yy_* will contribute to the out-of-plane shearing stress *τ_zx_*. Thus, the in-plane deformation of the (111) structure will inevitablely cause an out-of-plane deformation. This phenomenon can be verified by simulation. The modal simulation of the disk resonator using (111) silicon and a fully isotropic material were implemented, respectively. The results are depicted in Figure 7. The top view and side view of *n* = 2 wine-glass mode are demonstrated. There is an unbalanced out-of-plane deformation in the (111) disk resonator. However, no out-of-plane deformation was observed in the fully isotropic disk resonator. The ratio of the out-of-plane to the in-plane amplitutes are from 1:10 to 1:30, which depends on the structure height and beam width. This unbalanced out-of-plane deformation of the (111) disk resonator will cause considerable impact on the clamping loss.

Based on this, *Q* of the resonators made of (111) silicon will have an upper boundary. For disk resonators, it might be more favorable to use the (100) silicon. In this case, the *n* = 3 wine-glass mode is recommended instead of the *n* = 2 wine-glass mode, considering the mode-matching condition.

## 5. Conclusions

In this paper, the *Q* upper bond of the (111) disk resonator was demonstrated by engineering very low TED. It was experimental demonstrated that *Q* stops increasing when it reaches the value around 450,000. In this case, the clamping loss surpasses TED, become the primary damper. The unbalanced out-of-plane displacement caused by the coupling between the in-plane strain and out-of-plane stress was demonstrated. This indicates that the in-plane deformation of the (111) disk resonator could cause as much as 1/30 to 1/10 of out-of-plane deformation, which could cause considerable clamping loss.

## Abbreviations

The following abbreviations are used in this manuscript:

MEMS	Microelectromechanical systems
AC	Alternating Current
